# Exploring Post COVID-19 Outbreak Intradaily Mobility Pattern Change in College Students: A GPS-Focused Smartphone Sensing Study

**DOI:** 10.3389/fdgth.2021.765972

**Published:** 2021-11-23

**Authors:** Congyu Wu, Hagen Fritz, Melissa Miller, Cameron Craddock, Kerry Kinney, Darla Castelli, David Schnyer

**Affiliations:** ^1^Department of Psychology, University of Texas at Austin, Austin, TX, United States; ^2^Department of Civil, Environmental, and Architectural Engineering, University of Texas at Austin, Austin, TX, United States; ^3^Department of Diagnostic Medicine, University of Texas at Austin, Austin, TX, United States; ^4^Department of Kinesiology and Health Education, University of Texas at Austin, Austin, TX, United States

**Keywords:** COVID-19, college students, mobility pattern, smartphone sensing, GPS, principal component analysis, circadian rhythm, digital health phenotyping

## Abstract

With the outbreak of the COVID-19 pandemic in 2020, most colleges and universities move to restrict campus activities, reduce indoor gatherings and move instruction online. These changes required that students adapt and alter their daily routines accordingly. To investigate patterns associated with these behavioral changes, we collected smartphone sensing data using the Beiwe platform from two groups of undergraduate students at a major North American university, one from January to March of 2020 (74 participants), the other from May to August (52 participants), to observe the differences in students' daily life patterns before and after the start of the pandemic. In this paper, we focus on the mobility patterns evidenced by GPS signal tracking from the students' smartphones and report findings using several analytical methods including principal component analysis, circadian rhythm analysis, and predictive modeling of perceived sadness levels using mobility-based digital metrics. Our findings suggest that compared to the pre-COVID group, students in the mid-COVID group generally 1) registered a greater amount of midday movement than movement in the morning (8–10 a.m.) and in the evening (7–9 p.m.), as opposed to the other way around; 2) exhibited significantly less intradaily variability in their daily movement; 3) visited less places and stayed at home more everyday, and; 4) had a significant lower correlation between their mobility patterns and negative mood.

## 1. Introduction

Since the first cases of COVID-19 were confirmed in the United States in January 2020, organizations and individuals scrambled to come up with counter-measures to curb the spread of the virus. State and municipal authorities declared emergency or disaster status, issued stay-at-home orders, and canceled events. Colleges and universities around the country also started implementing decisive measures such as closing campuses and shifting instruction online. One direct consequence is the altered mobility patterns of nearly all people. The way in which people's daily routine changed was unprecedented. College students are a group of people especially influenced by the changes.

One way to assess mobility patterns is through the use of questionnaires or momentary assessment prompts (1). These methods have their limitations which could be overcome through the use of smartphone sensors, which provide an objective way to measure daily mobility behavior. College students would be an ideal population to examine this approach since they are heavy users of smartphones – carrying their smartphones with them everywhere they go. A number of smartphone sensing studies have been conducted to monitor health and behavior of college students (2, 3). Among smartphone embedded sensors, GPS data tracked over a continuous period of time is found to reflect fluctuations in daily movement patterns and used to detect other related behavior and health issues. We believe smartphone sensing is an appropriate approach to study the mobility change in college students due to the COVID-19 pandemic and the related societal response.

We collected smartphone sensing data from two groups undergraduate participants from the University of Texas at Austin to investigate mobility pattern change. One group was assessed from January to March, a period that resides mostly before the pandemic was officially declared. The second group was collected from May to August of 2020, during which the pandemic was in full force. To examine mobility and changes in mobility, we applied multiple analytic approaches—principal component analysis, circadian rhythm analysis, and digital phenotyping—to quantify the intradaily patterns of participants' movement. Existing studies tend to quantify mobility as a singular construct (e.g., the amount of movement) and the corresponding findings simply suggest “after the COVID outbreak that people traveled way less everyday.” We wanted to utilize more sophisticated GPS data processing techniques to tease out finer patterns that would reflect changes in mobility patterns across the entire 24-h period, rather than overall movement comparisons. The focus on college students, the use of mobile sensing to study mobility, together with the deep dive into the intradaily patterns are how this paper is unique in the current literature on COVID-19 related behavior change.

Our findings suggest that compared to the pre-outbreak group, students in the post-outbreak group 1) registered greater movements midday rather than in the morning (8–10 a.m.) or in the evening (7–9 p.m.); 2) exhibited significantly less intradaily variability in their daily movement; 3) visited less places and stayed at home more everyday, and; 4) had a significantly lower correlation between their mobility patterns and their depression symptoms. The first three findings portray an image of a less active and less organized day experienced by the post outbreak group. The last takeaway suggests that mental health prediction using personal sensing features is greater when participants' daily life has more normalcy, that is before the pandemic started.

## 2. Related Work

### 2.1. COVID-19 and Mobility Change

Researchers have sought evidence of COVID-19-induced mobility change from commercial mobile location data providers. Smartphone apps make location requests sporadically, resulting in a record of GPS locations registered by the smartphone. Commercial location data companies have collected anonymized location pings from an extremely large number of smartphones in the US and globally. An outstanding advantage of such mobile location data is its extensive geographic coverage, allowing researchers to aggregate the data based on specific regions of choice (e.g., city or county) and derive region-specific, population-level insights. The limitation is also significant. Because smartphone location pings are sporadic “snapshots,” the resulting data does not form a continuous portrait of the user's daily mobility pattern and thus cannot be used to understand individual-level behavior. We found several studies using data of this kind to evaluate population mobility change specific to geographic or administrative divisions. Using commercial mobile location data collected shortly after the COVID-19 outbreak, Warren et al. (4) showed a sharp decrease in population mobility in multiple major cities around the world. Gao et al. (5) mapped county-level mobility change in the United States and also found mobility decreases in the vast majority of counties in the early months of rapid COVID-19 spread. Engle et al. (6) discovered significant correlations between county-level mobility change and county-level infection rate and other socioeconomic indicators such as age and political affiliation. Couture et al. (7) investigated population movement between counties and states in the United States as well as visits to different types of commercial venues.

While the commercial data approach uncovers population level mobility patterns, a personal sensing approach proves useful to monitor individual participants' daily movement more closely, together with other behavioral aspects such as physical activity and sleep. Sun et al. (8) collected smartphone sensing (GPS, Bluetooth, phone usage; no accelerometer) and Fitbit data from a large, multi-national sample (1,062 participants) in Europe from early 2019 to mid 2020. They found significant decreases in daily distance traveled and the number of surrounding devices detected and increases in phone usage, sleeping time, and home stay after the COVID-19 outbreak. Sañudo et al. (9) collected smartphone sensing data (accelerometer and phone usage; no GPS) from 20 college students during two periods, once before and once during the COVID-19 lockdown and arrived at similar findings that are reduced physical activity, increased smartphone use, and longer sleeping hours. Most other studies investigating COVID-related behavioral changes in colleges students focused on constructs of physical activity and used questionnaire-based methods (10). To the best of our knowledge, no existing studies have used objectively measured location data to look into the intradaily patterns of college students as they experience the COVID-19 pandemic.

### 2.2. Mining GPS Data From Personal Sensing

Analytical methods for processing GPS data captured by smartphones and other smart wearable devices largely fall in two categories. Both approaches seek to convert the raw GPS trace captured over a period of time (e.g., a day) to a vector of feature values to feed as input to subsequent analyses such as predictive modeling or clustering. The first category is a raw data approach, which directly makes use of the raw GPS data collected and bulk-calculate descriptors or statistics using established algorithms or feature representation methods. This approach aims to preserve information contained in the raw GPS data and requires minimal researcher input on how to manipulate the data. One example of this approach is the vector space representation of GPS locations (11), which creates a location label (e.g., home, work, etc.) for every predetermined interval (e.g., 30 min) during the length of the GPS trace, thus forming a vector of labels characterizing the sequence of place types. The vector space method has also been used to represent other types of mobile sensor signals such as Bluetooth (12). Another example of this approach is training autoencoders using a displacement vector created by differencing the original GPS coordinate series (13). A key commonality of the methods that belong in this category is the division of a GPS trace into a series of sub-traces, thus preserving the characteristics of the original trace, and use simple measures of the sub-traces themselves as features without aggregation.

The second category is a feature engineering approach, which requires direct input from the researcher to devise metrics. Researchers have proposed various GPS features such as location variance, maximum distance covered, and percentage of time spent at home (14, 15), which can be further linked with behavioral and health outcomes and become *digital health phenotypes*. Because they are created with specific purposes to quantify specific constructs, these features are easily interpretable.

## 3. Method

### 3.1. Data

We used the Beiwe research platform (16) to collect smartphone sensing and real-time survey data from two groups of undergraduate students at the University of Texas at Austin (UT), over two non-overlapping periods in 2020. Beiwe is a mobile software suite through which smartphone sensor data and survey responses can be collected and uploaded to a server for research purposes. The study was approved by University of Texas at Austin IRB (study number 2019-09-0120). Participants underwent an initial screening before being consented into either phase of the study. Participants were only recruited if they were enrolled UT students between 18 and 35 years of age and had no current neurological and psychiatric/psychological disorders, current significant substance abuse, or hormone altering medication intake.

Enrollment for Phase 1 started in early January prior to the spring semester. The study period lasted from mid January to the end of March, corresponding to the period of time when the first cases of COVID-19 were confirmed in the US and no nation-wide counter measures had been taken. This study phase included 74 participants. All of the 74 participants had a primary residence in Austin during the study period. Enrollment interviews for Phase 2 were conducted over a period of 2 weeks with full enrollment completed by May 1st. The second phase of the study concluded when participants scheduled a virtual meeting with a study coordinator in late August to early September for an exit interview and to coordinate shipping study materials back to UT. Phase 2 consisted of 52 participants during which the pandemic and the related orders and mandates were in full effect. Twenty of the 52 participants lived in Austin and all of the 52 participants lived in Texas during the study period.

Smartphone sensing data we collected include GPS, accelerometer, and phone usage data from the participants' primary smartphones and real-time survey data includes participants' responses to daily activity, mood, and sleep questions. Specifically, the GPS data contain timestamped coordinates (longitude and latitude). The GPS sensor was configured to scan for 1 min every 10-min break, subject to hardware constraints such as phone power-off or user deactivating GPS. In total, we collected 6,442 days of GPS data across all 126 participants striding the two groups.

### 3.2. Experiments

We implemented three different methods to analyze smartphone GPS data collected from our participants in the pre- and post-outbreak groups: principal component analysis, circadian rhythm analysis, and digital phenotyping. The first two belong in the raw data approach whereas the third method involves feature engineering and predictive modeling.

#### 3.2.1. Principal Component Analysis

**Figure 1:**
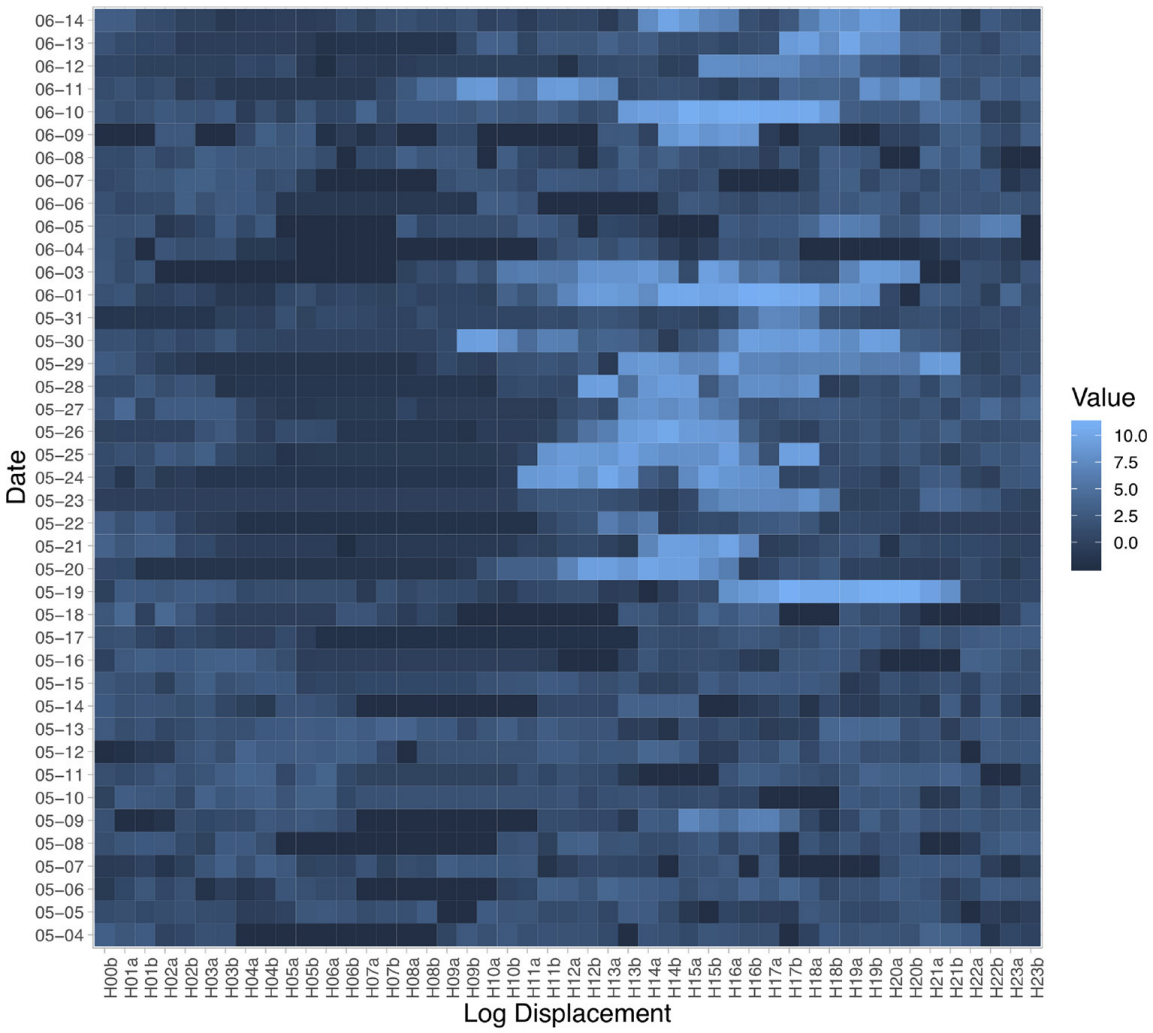
Daily Displacement Profile extracted from one participant's GPS data. Labels on the horizontal axis indicate the half-hour bins: for example, H06a indicates the 6-6:30 a.m. and H20b indicates 8:30-9 p.m. Cell color indicates the natural logarithm of the displacement detected by the smartphone GPS sensor during the current half-hour bin compared to the previous one. Brighter blue means greater displacement.

For Principal Component Analysis (PCA), we preprocessed the GPS data by constructing a Daily Displacement Profile for each day of each participant during which GPS data was collected. For each participant's each day's GPS data, we placed the entire GPS trace into 48 half-hour bins and calculate an average coordinate within each bin. Then we calculated the haversine distance between two adjacent bins and regard the vector of subsequent distance values as constituting a Daily Displacement Profile (DDP). A DDP consists of 47 displacement values because of the distance differencing. If during a half-hour bin, no GPS data was observed, then we carried over the coordinate from the most recent available location. Figure 1 shows the DDP of all the days collected from one example participant. Overall, we observe increased displacement values on some days during the day compared to in the early morning.

**Figure 2:**
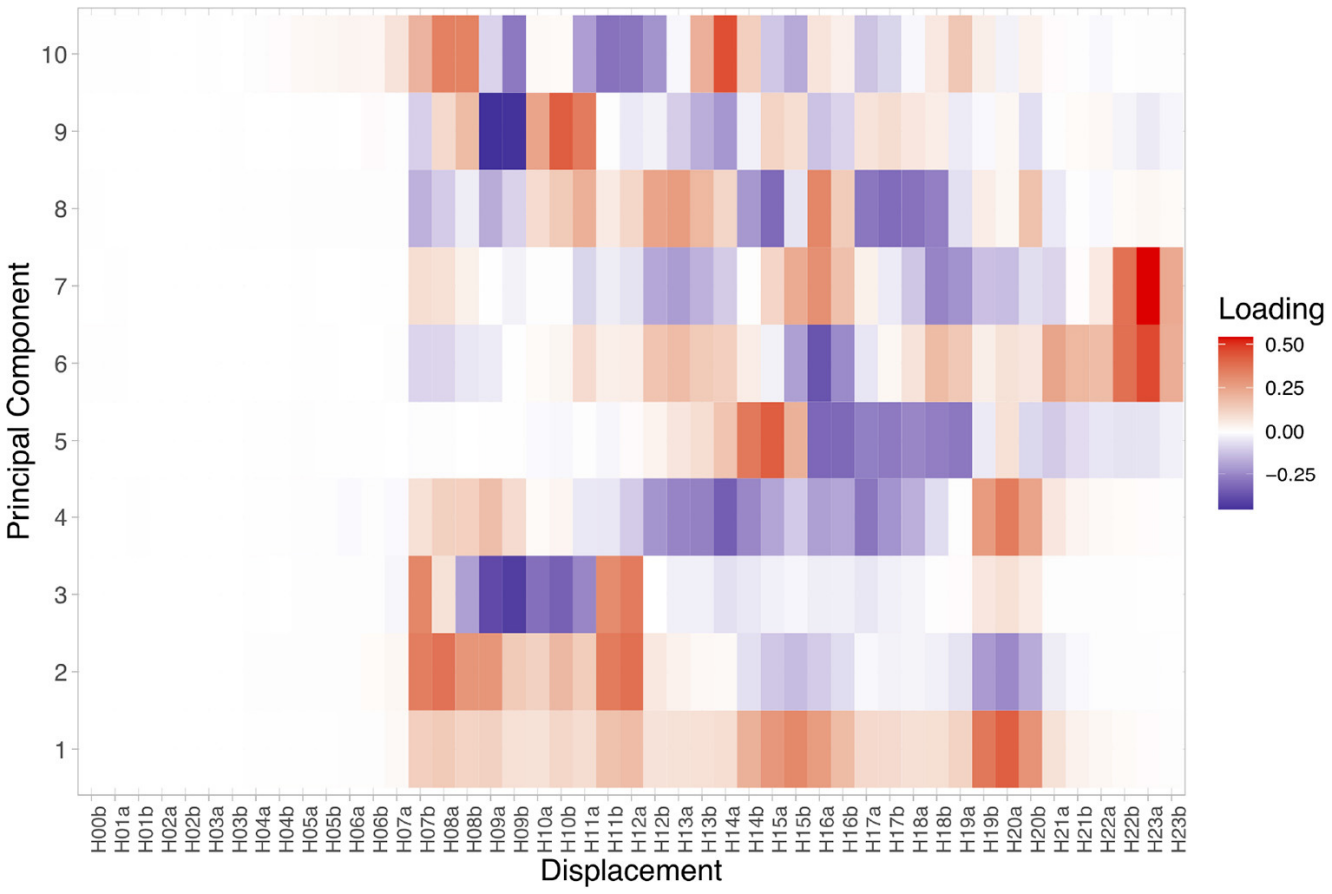
Loadings of the first 10 Principal Components of our participants' Daily Displacement Profiles. Labels on the horizontal axis are the same as Figure 1 and indicate the GPS displacement associated with each half-hour segment compared to the previous one. Cell color indicates the sign of a particular variable in relation to a PC. Red indicates that a specific PC is in the same direction as a variable whereas blue indicates that it is the opposite. Darker color (red or blue) indicates loadings with a greater absolute value.

Once the DDPs are constructed, we treat each dimension as a separate feature or variable and conduct PCA to discover the representative linear combinations of the local displacement values that can explain large proportions of the variance within the participants' movement patterns. The results indicate that 63% of variance is explained by the first 10 Principal Components (PCs); 81% of variance by the first 20 and 92% by the first 30. Figure 2 visualizes the weights on each displacement variable (i.e., loadings) of the first 10 PCs. The first Principal Component represents an entire day of moving around with two peaks at about 3 p.m. and 8 p.m. The second PC has positive weights on displacement in the morning hours but negative weights on displacement at around 8 p.m. All PCs had almost zero weights on the early morning hours, during which participants were most likely sleeping. We then performed the Welch *t*-test on the value of each of the first 10 PCs between the pre- and post-outbreak participant groups.

#### 3.2.2. Circadian Rhythm Metrics

Besides relative movement across different hours of the day, we were also interested in extracting circadian rhythm metrics from the participants' GPS data. Circadian rhythm quantifies the day-to-day regularity in the magnitude fluctuation of an individual's daily routine activity and is found to be correlated with many health and well-being statuses. We borrowed several important circadian rhythm metrics from the actigraphy analysis literature (17) and applied them on our GPS data. Specifically, we extracted the five metrics listed below using R package nparACT (17). Just like the DDP discussed in section 3.2.1, we first calculated each participant's GPS displacement for each 15-min bin during the study period and use the displacement values as signal magnitude. Here we chose a more granular binning strategy (15-min) in order to capture finer variations of daily movement.

- Interdaily Stability (IS): IS quantifies the stability of rest-activity rhythms or the invariability of the rhythm between different days. It takes a higher value when the daily distribution of a signal activity appears more similar across different days, hence more “stable.” IS is officially defined as $\frac{\sum_{h=1}^{24}{\left({\bar{X}}_{h}-\bar{X}\right)}^{2}/24}{\sum_{i=1}^{n}{\left(X_{i}-\bar{X}\right)}^{2}/n}$, where *n* represents the total number of sampled points, *X*_*i*_ is the *i*-th sampled signal magnitude, $\bar{X}$ is the mean value of all sampled points, and ${\bar{X}}_{h}$ is the mean value of sampled points within hour *h* ∈ {1, …, 24} across all days observed.
- Intradaily Variability (IV): In contrast to IS, IV quantifies the fragmentation of a rest-activity pattern within each day. It takes a higher value when signal strength fluctuates consecutively between high and low more intensively during each day. IV is officially defined as $\frac{\sum_{i=2}^{n}{\left(X_{i}-X_{i-1}\right)}^{2}/\left(n-1\right)}{\sum_{i=1}^{n}{\left(X_{i}-\bar{X}\right)}^{2}/n}$, following the same notation above for IS.
- M10: the average magnitude of the signals (i.e., displacement) over the 10 consecutive hours that have the maximum signal magnitude over all days observed.
- L5: the average magnitude of the signals (i.e., displacement) over the five consecutive hours that have the minimum signal magnitude over all days observed.
- Relative Amplitude (RA): defined by $\frac{M10-L5}{M10+L5}$, increases when there is a sharp contrast between the active and inactive periods within each day. A uniform distribution of signal magnitude (i.e., no concrast between active and inactive periods) would result in a RA of 0.

These metrics are again daily metrics. Similar to our analysis on PCs, we performed the Welch *t*-test on the value of each of the five circadian rhythm metrics between the pre- and post-outbreak participant groups.

#### 3.2.3. Digital Phenotyping

The previous two methods were based on Daily Displacement Profiles. Additionally, we explored the feature engineering approach and extracted seven daily-level digital phenotypes from our participants' GPS data:

- Location variance (loc.var): square root of the variance in GPS coordinates.
- Number of places visited (num.pls): determined by an established temporal clustering algorithm (18). The algorithm utilizes the distance and time difference between consecutive GPS coordinates and segments a GPS trace into periods of staying at different places and periods of transition between places. The number of distinct places extracted serves as the value of this feature.
- Normalized entropy of time spent at different places visited (ent.pls): greater values indicate more equally distributed time spent at different places extracted, whereas lower values indicate most time spent at a small number of places.
- Percentage of time spent at home (perc.home): home is defined by the place extracted where a participant spent the most time between 12 and 6 a.m. during the study period.
- Total distance traveled (total.dist): the sum of the distance between every pair of consecutive GPS coordinates registered.
- Maximum distance covered (max.dist): the greatest distance between any two GPS coordinates registered.
- Routine index (routine.idx): quantifying the degree to which a participant's mobility pattern over a period of time is similar to that of the same period of time on all days, as formulated by Canzian and Musolesi (14).

With these daily GPS features computed for all participants in both groups, we further carried out two analyses. First, like the Principal Components and Circadian Rhythm metrics, we compared the values of these daily between the pre- and post-outbreak group using the Welch *t*-test. Second, we built supervised learning models using these GPS features to predict the experience of severe sadness during the concurrent day. Daily sadness experience was solicited from participants in both groups via smartphone delivered surveys in the study. At a random time during the day, we presented participants with the prompt “right now I am feeling __________ sad” with answer options “not at all,” “a little bit,” “quite a bit,” and “very much” to choose from. We consider an observation as *severe sadness* when the self-reported sadness level is “quite a bit” or “very much” because these two levels would justify intervention. While correlating personal sensing data (especially mobility patterns characterized by GPS traces) with mood experience or mental health symptoms is a typical practice in digital health phenotyping (19, 20), our objective here is to discover whether there exists a significant performance discrepancy between the two groups, since the post-outbreak participants were under influence by the pandemic and subject to systematically altered mobility patterns. We limited the data used for the predictive modeling of severe sadness to those participants who reported being at least “quite a bit” sad at least twice over their study period (pre or post) because there is little point in predicting severe sadness for those who are never affected by it. Thirty-five out of the 74 participants in the pre-outbreak group, and 27 out the 52 participants in the post-outbreak group were thus retained for the analysis. We extracted GPS features from the 24-h period leading up to each sadness self-report and used two machine learning methods to evaluate performance of predicting severe sadness: 1) mixed-effect logistic regression with random participant effect and 2) random forest with leave-one-out cross validation per participant. Logistic regression is a typical approach for two-class classification problems and random forest is a popular, high-performing machine learner for many supervised learning tasks including in digital health phenotyping applications (21).

## 4. Results

### 4.1. Principal Component Analysis

**Figure 3:**
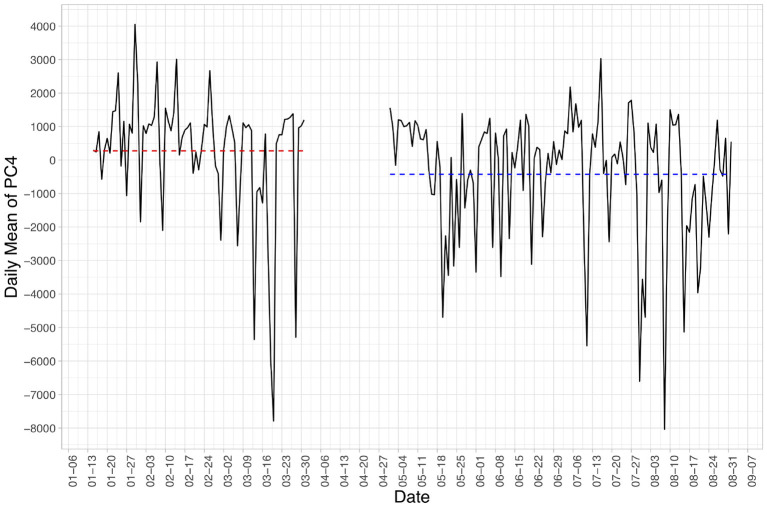
Daily mean value of Principal Component No.4 (PC4) over the two study periods. The red and blue dashed lines indicate the average value from the pre- and post-outbreak group, respectively.

Out of the first ten PCs extracted from our participants' Daily Displacement Profiles, one PC—the fourth Principal Component (PC4)—turns out to be significantly different between the pre- and post-outbreak group. PC4 is significantly lower (*p* < 0.001; −322.4 post vs. 406.3 pre) in the post-outbreak group than the pre-outbreak group. The remaining nine PCs do not achieve statistical significance. We aggregated the value of PC4 by its mean value by the day to show its variation throughout the two study periods (Figure 3). The lower value of PC4 in the post-outbreak group is visible with more days below zero than the pre-outbreak group.

As shown in Figure 2, what PC4 represents is a day with increased movement in the morning (~8 a.m.) and in the evening (~8 p.m.) but decreased movement in-between. As such, participants in the post-outbreak group spent more of their days going places during the day whereas participants in the pre-outbreak group had a more concentrated mobility pattern with greater movement in the morning and in the evening.

### 4.2. Circadian Rhythm Metrics

Out of the five Circadian Rhythm metrics described in section 3.2.2, Intradaily Variability (IV) and Relative Amplitude (RA) showed statistical significance between the two groups whereas the remaining three did not. The post-outbreak group is significantly lower in both IV (*p* = 0.004) and RA (*p* = 0.002) than the pre-outbreak group. Of note, Interdaily Stability (IS), characterizing the variation in circadian rhythm over multiple days, is not significantly different between the two groups.

Straightforwardly, a lower IV indicates less presence of rest-activity alternation within a day, and a lower RA indicates a smaller contrast between the magnitude of movement between active and inactive periods within a day. Significant lower values in both IV and RA suggest that participants experienced less “chaotic” days in terms of mobility in the post-outbreak group.

### 4.3. Digital Phenotyping

**Figure 4:**
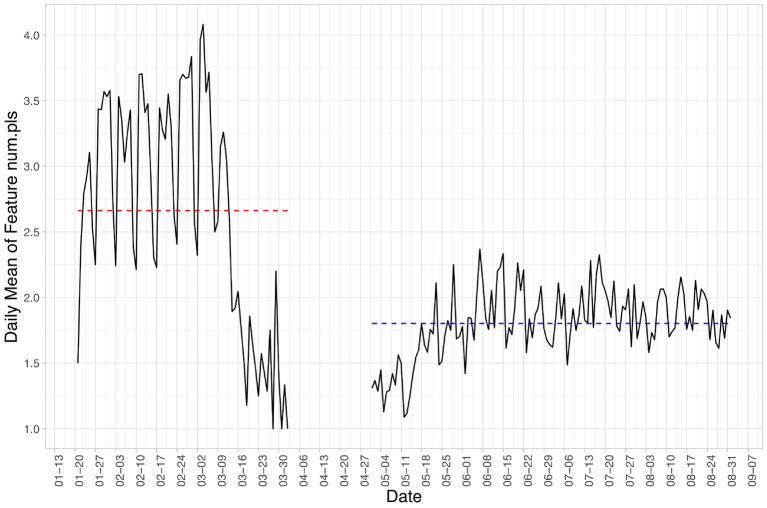
Daily mean value of the *num.pls*, number of significant places visited, over the two study periods. The red and blue dashed lines indicate the average value from the pre- and post-outbreak group, respectively. Note the drastic drop in March in the pre-outbreak group, coinciding with the declaration of pandemic in the US on March 13, 2020.

Out of the seven GPS features described in section 3.2.3, three features were significantly different between the two participant groups, namely *num.pls* (number of significant places), *ent.pls* (normalized entropy of time spent at significant places), and *perc.home* (percentage of time spent at home). Of the three, *num.pls* and *ent.pls* were significantly lower in the post-outbreak group with *p*-values both lower than 0.001. Participants in the post-outbreak group on average visited one less significant place during their days compared to their pre-outbreak counterparts [1.73 post vs. 2.73 pre, illustrated on a day-to-day basis in Figure 4; note the drastic drop in March in the pre-outbreak group, coinciding with the declaration of pandemic by the then-president of the United States on March 13, 2020 (22)]. The percentage of time spent at home, on the other hand, is significantly higher in the post-outbreak group than the pre-outbreak group (20.8% post compared to 16.3% pre). These contrasts suggest that in the post-outbreak group participants were more home-bound and ventured outside less than before the pandemic.

As for performance of the predictive modeling tasks targeting severe sadness experience, the area under ROC value for the pre-outbreak group was 0.71 with a standard deviation of 0.13 whereas it was 0.68 for the post-outbreak group with the same standard deviation of 0.13. The direction of this difference is expected because we hypothesized that due to systematically altered mobility pattern in the post-outbreak group, the pre-established (by studies conducted during normal times) correlation between mobile sensed mobility pattern and mental health symptoms should be lower. However, the difference is not statistically significant in our experiment (*p* = 0.38), possibly due to the relatively small number of participants tested upon.

## 5. Discussion

Some of our findings such as the significantly increased at-home time in the post-outbreak group is consistent with findings in similar studies with entirely different participant cohorts (8). Compared to current literature, our findings offer new insights into the intradaily mobility patterns of college students during the pandemic, such as the reduced number of significant places visited during a day, the reduced Relative Amplitude in daily displacement profile from our circadian rhythm analysis, and the shifted temporal distribution of daily movement over different hours as revealed by our Principal Component Analysis.

One limitation of our study is the potential confounding factor that is the eventual overlap between summer time and the timeline of the post-outbreak study period. Without data from a similar period pre-COVID, it is difficult to know what the “normal” pattern of activity change would be, between a college semester and summer break. However, with generally fewer constraints of academic activities, college students might tend to be more active during summer time. If this is the case, then we believe our findings regarding reduced movement in our post-outbreak group would accentuate the effect of the pandemic on student mobility patterns.

## 6. Conclusion

In this paper we presented our findings from a two-period smartphone sensing study we conducted using college student participants at a major US public university before and during the COVID-19 pandemic. We focused on the mobility patterns revealed by the GPS data collected from the students' smartphones and applied three analytical methods, namely principal component analysis, circadian rhythm analysis, and digital phenotyping, to characterize the differences in intradaily movement patterns between the two groups. Our findings suggest that compared to the pre-COVID group, students in the mid-COVID group 1) registered significantly more movement *during* the day rather than in the morning (8–10 a.m.) and in the evening (7–9 p.m.); 2) exhibited significantly less intradaily variability in their daily movement; 3) visited less places and stayed at home more everyday, and; 4) had a significant lower correlation between their mobility patterns and their depression symptoms. These findings together portray a less active, less structured, and more home-bound daily movement routine of college students in the post-outbreak group and deepen our understanding of the ways college students' daily lives have been affected by the COVID-19 pandemic.

## Data Availability Statement

The data used in this study is available upon request with permission of the University of Texas at Austin. Requests to access these datasets should be directed to Congyu Wu, congyu.wu@austin.utexas.edu.

## Ethics Statement

The studies involving human participants were reviewed and approved by University of Texas Social and Behavioral IRB. The patients/participants provided their written informed consent to participate in this study.

## Author Contributions

CW performed the statistical analyses and wrote the first draft of the manuscript. HF and DS wrote sections of the manuscript. MM handled IRB compliance, participant recruitment, and logistics of data collection. All authors contributed to conception and design of the study and contributed to manuscript revision, read, and approved the submitted version.

## Funding

This work was supported by Whole Communities—Whole Health, a research grand challenge at the University of Texas at Austin.

## Conflict of Interest

The authors declare that the research was conducted in the absence of any commercial or financial relationships that could be construed as a potential conflict of interest.

## Publisher's Note

All claims expressed in this article are solely those of the authors and do not necessarily represent those of their affiliated organizations, or those of the publisher, the editors and the reviewers. Any product that may be evaluated in this article, or claim that may be made by its manufacturer, is not guaranteed or endorsed by the publisher.
